# Arthroscopic partial meniscectomy in middle-aged patients with mild or no knee osteoarthritis: a protocol for a double-blind, randomized sham-controlled multi-centre trial

**DOI:** 10.1186/1471-2474-14-71

**Published:** 2013-02-25

**Authors:** Kristoffer B Hare, L Stefan Lohmander, Robin Christensen, Ewa M Roos

**Affiliations:** 1 Institute of Sports Science and Clinical Biomechanics, University of Southern Denmark, Odense, Denmark; 2 Department of Orthopedics, Clinical Sciences Lund, University of Lund, Lund, Sweden; 3 Department of Orthopedics and Traumatology, University of Southern Denmark, Odense, Denmark; 4 Musculoskeletal Statistics Unit, the Parker Institute, Department of Rheumatology, Copenhagen University Hospital, Frederiksberg, Copenhagen, Denmark

## Abstract

**Background:**

Arthroscopic partial meniscectomy has been shown to be of no benefit to patients with concomitant knee osteoarthritis, but the optimal treatment of a degenerative meniscus tear in patients with mild or no knee osteoarthritis is unknown. This article describes the rationale and methodology of a randomized sham-controlled trial to assess the benefit of arthroscopic partial meniscectomy of a medial meniscus tear in patients with mild or no knee osteoarthritis. The objective of the study is to test whether the benefit from arthroscopic partial meniscectomy in patients with knee pain, medial meniscus lesion and mild/no knee osteoarthritis, is greater after arthroscopic partial meniscectomy than following sham surgery.

**Methods:**

We will conduct a randomized controlled trial of treatment for degenerative meniscus tears in middle-aged patients (aged 35–55 years) with an MRI-verified medial meniscus lesion and mild or no knee radiographic osteoarthritis (grade 0–2 on the Kellgren & Lawrence scale). Patients will be randomized to receive either conventional arthroscopic partial meniscectomy or a sham surgery procedure. The primary outcome will be the KOOS_5_ derived from the ‘*Knee Injury and Osteoarthritis Outcome Score’* at 2 years follow-up. Secondary outcomes at 2 years will include all five individual subscales of the KOOS, a global perceived effect score, the Short-Form-36 health status score, EQ-5D for economic appraisal and objective tests of muscle strength and physical function. Radiographic knee osteoarthritis will be evaluated at 5 years.

**Discussion:**

Demonstration of no additional benefit from arthroscopic partial meniscectomy on pain and function should lead to a change in clinical care of patients with a degenerative meniscus tear. The results of this study will provide empirical evidence for the potential benefit/harm of arthroscopic partial meniscectomy compared to a masked sham-therapeutics intervention.

**Trial registration:**

NCT01264991

## Background

A degenerative meniscus tear can be both a risk factor for knee osteoarthritis (OA) and a sign of disease [1]. The current standard treatment for a degenerative meniscus tear is arthroscopic partial meniscectomy (APM), the most commonly performed orthopedic procedure, carried out on 1 million patients annually in the USA [2]. Both meniscus injury and a meniscectomy are associated with the development of knee OA [3-5]. Previous studies have found APM to be no better than, or have no additional benefit in comparison to, sham surgery, lavage, optimized non-surgical treatment, or exercise [6-8]. In all these studies, patients with knee OA were included and the mean ages ranged from 52 to 62 years. The benefit in a younger population from an APM procedure in a knee with a degenerative meniscus tear and mild or no knee OA is, however, uncertain and needs to be further investigated.

The benefits of the APM procedure on pain and function in patients with a degenerative meniscus tear, were firstly described in non-controlled studies from the 1980s to the 1990s [9-11] when arthroscopic procedures were gaining acceptance. However, poorly designed studies (retrospectively, use of non-validated outcome measures, small patient populations, lack of control groups and randomized allocation) have prevented firm conclusions about the effect of the APM procedure. A previous, but now withdrawn, Cochrane review from 2000 [12] concluded: *”…lack of randomized trials means that no conclusions can be drawn on the issue of surgical versus non-surgical treatment of meniscus injuries”.* At the time of this submission, there are, to our knowledge, no published high quality controlled, randomized studies that show a benefit from APM as compared to other treatment modalities (placebo, physiotherapy, medication or exercise) on pain and function in patients aged 35–55 years with a degenerative medial meniscus tear.

Both meniscus injury and meniscectomy are associated with a high risk of knee OA. Surgical resection of the meniscus leads to increased joint cartilage contact stress through altered load transmission, decreased shock absorption, and decreased joint stability [13]. Of patients who undergo either total or partial meniscectomy, 50% on average develop knee OA within 10–20 years [5]. In the elderly population in general, and in patients with radiographic knee OA but no surgery, there is a higher incidence of MRI-verified concomitant meniscus injury compared to controls [4]. It remains unclear though, whether meniscectomy increases the risk of knee OA *per se*, compared to non-surgical treatment of a meniscus injury. In this study, incidence or progression of radiographic OA will be assessed at 5 years.

## Methods

### Study design

The study is designed as a prospective double-blind randomized sham-controlled, multi-centre trial (RCT). Patients will be randomly allocated to receive either an APM or sham (i.e. placebo) procedure. The study is designed according to current international standards and will be reported using the recommendations in the CONSORT statement [14]. The study is approved by the Research Ethics Committee of Region Zealand, Denmark, and is consistent with the Declaration of Helsinki.

Eligible patients will be screened using standardized fixed flexion radiography of both knees, to assess the degree of radiographic knee OA. If no, or at most mild, knee OA on radiographs (Kellgren & Lawrence grades 0–2) is present, written information about the study and a 10-minute information video will be given to the patients to view at home. They will also be handed a Patient-Reported Outcomes (PRO) questionnaire to fill out at home to minimize bias. At the second contact, the patients will receive an MRI scan of the affected knee and perform tests of physical function. Thereafter, the relevant researcher and the patient will be informed of the MRI findings. If the MRI confirms a medial meniscus lesion, the patient will be invited to participate in the study. Patients not consenting to randomization will be followed as an observational cohort with consecutive PRO evaluation at the same time points as those included in the RCT. However, the observational cohort will not be part of the Intention-To-Treat (ITT) population.

**Figure 1:**
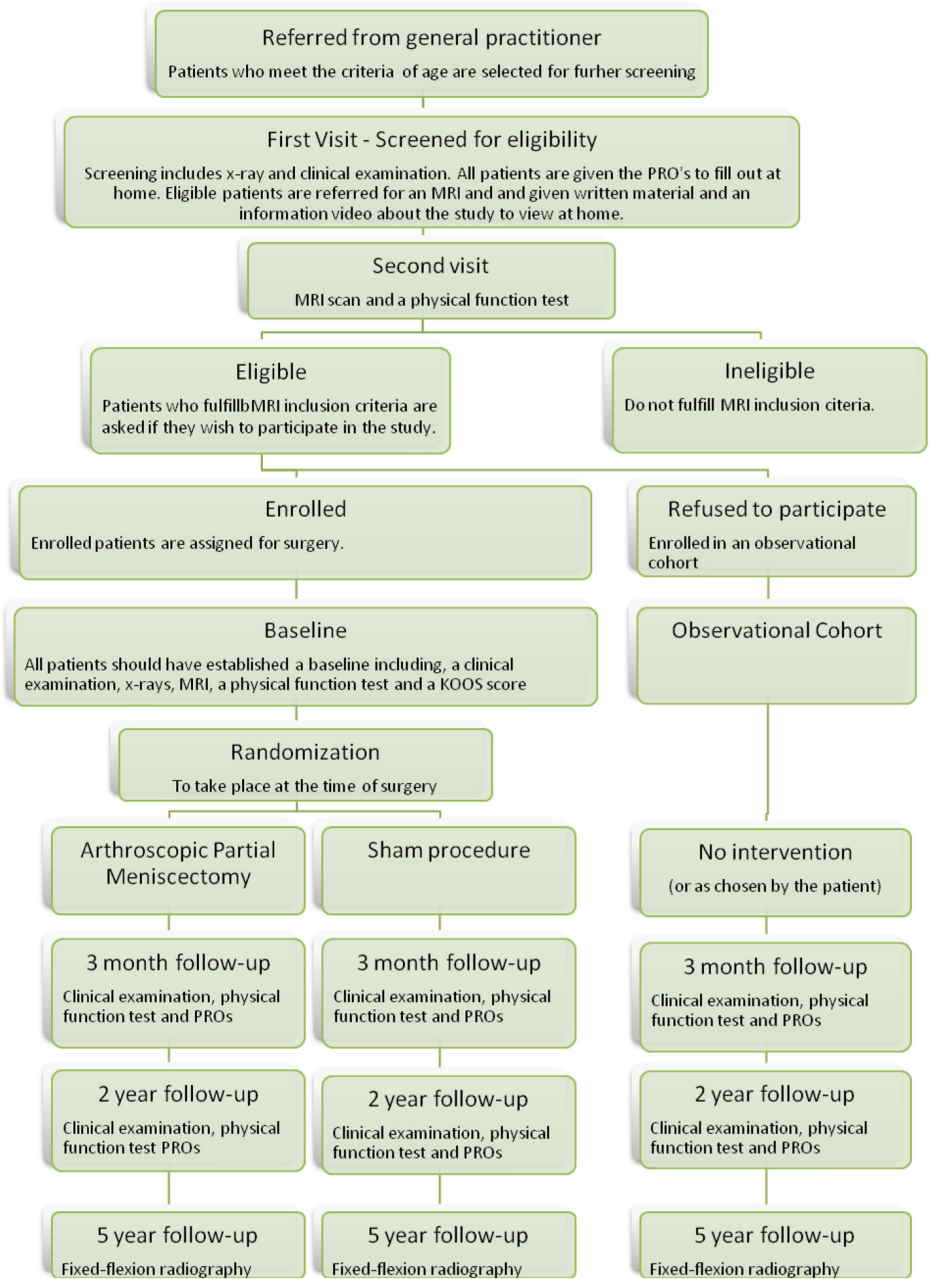
Study flowchart.

At 3 months, patients will have a clinical examination, fill out PRO questionnaires, and perform objective tests of muscle strength and physical function. At 2 years, follow-up will take place under the same conditions. At 5 years, all patients will have radiography of their knees to assess possible onset or progress of knee OA from baseline. The flowchart provides a visual description of the study (Figure 1).

### Purpose and hypothesis

The purpose of the present study is to determine whether the benefit from arthroscopic partial meniscectomy in patients aged 35–55 years with knee pain and an MRI-verified medial meniscus lesion, is greater after arthroscopic partial meniscectomy than following sham surgery. In addition, a 5-year follow-up of the same cohort will compare the effect of meniscectomy or sham surgery on the incidence and progression of radiographic knee OA.

We hypothesize that at 2 years, the change (improvement) in KOOS_5_, a composite score derived from the five subscales of the KOOS, is no greater after APM than following sham surgery. Further, we hypothesize, that at 5 years the rate of radiographic knee OA incidence and progression is greater in the APM group than in the sham surgery group.

### Participants

Eligibility criteria are patients between 35 and 55 years of age with knee pain for more than 2 months without significant trauma and an MRI-confirmed medial meniscus lesion. The patients must be eligible for outpatient surgery. Patients will be excluded if they are in need of acute surgery e.g. locking knees or high-energy trauma. Patients with grade 3 or 4 knee OA on the Kellgren & Lawrence classification [15,16] or knee surgery within the previous 2 years will also be excluded. Patients must be able to speak Danish and be free of any drug or alcohol abuse. Also, patients with trombophilia are excluded so as to prevent a high risk of deep venous thrombosis. The patients will be recruited through outpatient departments of the orthopedic clinics in Region Zealand on referral from general practitioners.

### Interventions

Patients will be randomized to receive either arthroscopic partial meniscectomy (intervention A) or placebo procedure/sham surgery (intervention B).

### Intervention A

The arthroscopic partial meniscectomy will be performed on an outpatient basis by experienced surgeons who are at least in their final year of residency or are attending orthopedic surgeons. We expect between 5–10 surgeons to be involved in the study. All arthroscopies will be performed with general anesthesia combined with local anesthesia (Bupivacain combined with Adrenalin) 20 + 20 ml extra- and intra-articularly, respectively. After general anesthesia is induced, the knee will be examined for stability. Thereafter, two standard portals on the lateral and medial sides of the ligamentum patella will be created but no outflow cannula inserted. An arthroscope will be used with a pressure-controlled irrigation system. Tourniquet use will be at the discretion of the surgeon. The strategy for the meniscectomy will be to preserve as much tissue as possible. A standard operation protocol will be used to document possible findings in cartilage, ligaments, synovium and the medial and lateral menisci. The type, and extent of meniscus lesion will be registered and changes in the articular cartilage will be classified according to the ICRS classification [17].

### Intervention B

The sham procedure will be performed under the same conditions as the arthroscopic surgery (Intervention A). In summary, the patient will be fully sedated with general anesthesia and the stability of the knee will be examined. Local anesthetic will be applied and two skin incisions will be made at the same locations and of the same size as in Intervention A. Then the knee will be manipulated as if a real arthroscopy was performed, the spillage of water and all other equipment needed for an arthroscopy will be used. A pre-recorded video of a standard arthroscopic partial meniscectomy will be played during the procedure. No instruments will enter the arthroscopy portals to avoid the possibility of deep infection, osteochondral lesions or unwanted interventions by the surgeon.

### Postoperative regime (independent of concealed group allocation)

All patients in both intervention groups will be given a folder including an exercise program for postoperative patients after knee arthroscopy. The folder gives a presentation of seven different non-weight bearing exercises (for the first week after surgery) and a further three weight-bearing exercises thereafter. The exercises are for the patients to carry out at home. The patients are also recommended to start unloaded cycling, swimming or walking after 1 week, and jogging or loaded cycling after 2–3 weeks.

### Primary outcome

All outcomes are listed in Table 1. The primary outcome at 2 years follow-up will be KOOS_5_, a composite score derived from the *Knee injury and Osteoarthritis Outcome Score* (KOOS) [18,19]. The KOOS is a self-reported questionnaire comprising five subscales: pain, other symptoms, activities in daily living (ADL), function in sport and recreation and knee-related quality of life (QOL). The previous week is taken into consideration when patients are answering the questions. Standardized answer options are given (5 boxes on a Likert scale) and each question gets a score from 0 to 4. A normalized score (100 indicating no symptoms and 0 indicating extreme symptoms) is calculated for each subscale.

**Table 1:** Summary of measures to be collected

**Variable**	**Baseline**	**Intermediate**	**Primary endpoint**	**Follow-up**
	**T = 0 mths**	**T = 3 mths**	**T = 2 yrs**	**T = 5 yrs**
***Baseline data***				
Age – yr	@	n.a.	n.a.	n.a.
Female sex - no. (%)	@	n.a.	n.a.	n.a.
Duration of knee symptoms - months	@	n.a.	n.a.	n.a.
Height - cm	@	n.a.	n.a.	n.a.
Body weight - kg	@	n.a.	@	@
Self-efficacy scale	@	n.a.	n.a.	n.a.
***Knee injury and Osteoarthritis Outcome Score (KOOS)***				
Pain – range: 0-100	@	@	@	@
Symptoms - range: 0-100	@	@	@	@
Function in daily living – range: 0-100	@	@	@	@
Function in sport and recreation – range: 0-100	@	@	@	@
Knee related Quality of life – range: 0-100	@	@	@	@
***EQ-5D***				
Global disease descriptive system	@	@	@	@
VAS patient global assessment of disease status - 0-100	@	@	@	@
***Short-Form-36 health survey - acute form***				
Physical component summary – range: 0-100	@	@	@	@
Mental component summary – range: 0-100	@	@	@	@
***Global Perceived Effect***				
7 step scale ranging from much worse to much better	n.a.	@	@	@
***Standardized knee radiographic with SynaFlexer***				
JSW and presence of osteophytes	@	n.a	n.a	@
***Performance measures***				
Single leg hop test	@	@	@	n.a.
Knee-bending test	@	@	@	n.a.
Isometric knee extensor strength	@	@	@	n.a.

@ = Assessed; n.a. = not assessed; VAS = Visual Analogue Scale; JSW = joint space width.

Subsequently, KOOS_5_ is calculated as a mean of the 5 subscale scores [KOOS_pain_ + KOOS_symptoms_ + KOOS_ADL_ + KOOS_sport &rec_ + KOOS_QOL_]/5.

### Secondary outcomes

#### KOOS

All five subscales from the KOOS will be included individually as secondary outcomes to support a clinically valid interpretation of the result.

#### Global perceived effect

All patients are asked to answer on a seven-step global rating scale (ranging from much worse, worse, slightly worse, no difference, slightly better, better to much better) the overall improvement in their knee symptoms after the operation. This is implemented to determine the minimal important change in the PROs. A clinically important change is considered when the patient reports an improvement or worsening of at least 2 steps from ‘no difference’, corresponding to ‘better’ or ‘worse’ on the scale [20].

#### Generic patient reported outcomes

Scores on the Medical Outcomes Study 36-item Short-Form General Health Survey (SF-36) [21,22], which reflect the health-related quality of life (SF-36 Health Survey) – Acute version (1 week re-call period) will be used as a generic measure of patient health status at 3 and 24 months. The SF-36 is comprised of 8 single subscale scores associated with physical and mental health.

The Euroqol 5 Dimension (EQ-5D) health score will be evaluated at baseline and at 3 and 24 months as a generic measure for economic appraisal [23,24]. EQ-5D consists of two pages - the EQ-5D descriptive system and the EQ visual analogue scale (EQ VAS). The EQ-5D descriptive system comprises the following 5 dimensions: mobility, self-care, usual activities, pain/discomfort and anxiety/depression. Each dimension has 3 levels: no problems, some problems, severe problems.

#### Performance measures

Tests of physical function will be performed at baseline and after 3 and 24 months including a one-leg jump test, maximum number of knee bends in 30 seconds and an isometric knee extension strength measurement. The patients will wear shorts, t-shirts, and sneakers. Tubigrip stockings will cover both knees to disguise scars from surgery; i.e., the test leaders will be masked regarding injured knee. In order to avoid bias from the effect of learning, randomization will be performed at each visit to determine which leg is to be tested first.

#### Single leg hop test

The one-leg hop will be included as a measure of physical function at a level above activities of daily living [25]. The one leg hop requires leg muscle strength, knee stability and confidence in knee function [26]. Subjects will perform two practice trials and then three test trials on each leg with hands behind their back. The best of the three test trials will be used. An additional trial will be performed if the patients improve more than 10 centimeters from trial two to trial three [27].

#### Knee-bend test

The maximum number of knee-bends performed in 30 seconds will be included as a measure of one-legged physical function required in daily life. This test requires fast changes between concentric and eccentric work and resembles stepping down a stair and is valid and reliable in meniscectomized patients [27].

#### Isometric knee extensor strength

Maximum knee-extension force will be measured using a hand-held dynamometer (Powertrack Commander). Patients will sit at the end of the examination couch with hip angle at 90° and knee angle at 60°. A large Velcro strap will be attached to the examination couch and the patient’s ankle will be perpendicular to the lower leg. The transducer will be placed at the front of the ankle under the Velcro strap to measure knee extension force. Patients will be instructed to contract “as forcefully as possible” with a gradual increase in force and strong verbal encouragement will be provided during the contractions. They will perform 3 contractions separated by a 60-second pause, and the highest value will be used as the result.

The reliability of the isometric muscle tests with a hand-held dynamometer has been reported to be satisfactory [28-30]. The knee extension strength will be expressed as maximal voluntary torque per kilo of body mass using the external lever-arm length and body mass of each patient.

#### Radiographic OA

To evaluate progression of knee OA, a fixed-flexion radiography procedure, with use of SynaFlexer [31], will be performed at baseline and after 5 years. This provides radiography at the exact same position and has been validated in determining joint space width (JSW) in knee osteoarthritis [32]. A single reader will score all the study films from baseline and 5-year follow-up and will be blinded to all clinical and questionnaire data and the baseline x-ray result but not to the sequence of the x-rays. A score will be assigned to each x-ray based on JSW and presence of osteophytes using a standard atlas [33].

### Exploratory outcomes

A questionnaire of patient self-efficacy modified from the Danish Arthritis Self-Efficacy Scale to suit this somewhat younger age group (not formally validated) and a question on patient expectations will be included. Demographic data will also be collected. Furthermore, participating patients will be asked two questions regarding their study participation. 1) “Which reason is the most motivating for your participation?” and 2) “Which information was the most useful when deciding?” Physical therapy prescribed by either a general practitioner or a research staff member will be carefully monitored with regard to the number of exercise sessions.

### Adverse events

Adverse events (not necessarily implying causality) will be registered in both treatment arms. A priori defined adverse events are: superficial infection, nerve or vessel injury, deep infection, compartment syndrome, deep venous thrombosis, myocardial infarction, stroke, and death. Re-arthroscopy is also considered an adverse event. Adverse events will be gathered from patients themselves, from the patient record review, and from the Danish National Patient Index (NPI) at the 3 and 24 months follow-ups.

### Sample size

The sample-size calculation is based on the assumed superiority of the arthroscopic procedures over the sham procedure. For a two-sample pooled t-test of a normal mean difference with a two-sided significance level of 0.05, assuming a common standard deviation (SD) of 15 in the KOOS_5_ score, a sample size estimation of the ITT population indicated that 36 individuals per group would be required to obtain a power of at least 80% to detect a minimal important change (MIC) of 10 KOOS_5_ score units. The MIC of 10 points and SD of 15 is based on findings from similar patient groups and interventions [19].

Following these estimations, it was decided to include 80 individuals in total (40 patients in each group), allowing for a 10% drop-out rate.

### Randomization

We will generate the two comparison groups using simple randomization, with an equal allocation ratio (1:1), by referring to a computer-generated table of random numbers. To ensure an equal distribution in the two groups, we will use a block randomization, using blocks of 4 and 6. Participants will be stratified for treatment centre. To ensure concealment of the assigned intervention, the surgeon will obtain a sealed envelope containing the participant’s assigned intervention after the patient is in the operating suite and has been fully sedated. The consecutively numbered envelope will be retrieved from a briefcase located at the actual operating theatre. The above mentioned allocation sequence will be generated by an external co-investigator, the enrolment will be performed by the first author and the assignment will be at the operating room where the envelope will be opened by the surgeon.

### Blinding

The RCT will be a double-blind trial. All study personnel and participants will be blinded to the intervention, except for the surgeons and other operating theatre personnel, who do not have any other role in the study.

### Statistical methods

Treatment groups will be examined for comparability at baseline with respect to demographic and prognostic factors. An ITT analysis based on all the randomized individuals - for the efficacy measures - will apply. Comparisons between groups of the primary end point will include all repeated measures and be analyzed with the use of a mixed effects model, with random factors for participant and centre.

Clinically important or relevant difference for the KOOS_5_ and KOOS subscales were chosen as 10/100 points. Thus a confidence interval excluding differences greater than 10 units between groups will be interpreted as indicating the absence of a clinically meaningful difference. This means that, if the 95% Confidence Interval around the group mean difference does not include a *potential* clinical benefit of 10 KOOS points, then we will then consider the therapeutic strategies equal.

Patients in the sham group who, later during the course of the study, may have an APM procedure will, according to the ITT principle, still be analyzed in the group to which they were originally allocated. Secondarily, all analyses will be supported/interpreted in the context of the corresponding results according to the per protocol populations.

### Treatment failure

No a priori criteria for cross-over are given. Should a patient contact the department because of unbearable symptoms they will be un-blinded and in case of having had placebo surgery they will be offered a new arthroscopy. These patients will be treated as cross-overs and still be included in the study. In case of the patient having had arthroscopic surgery in the first place the patient will be referred to the responsible surgeon who will be in charge of referring to further surgical or non-surgical treatment and/or investigation. Both patient groups will be asked to fill out a KOOS questionnaire at the extra visit and will continue to be followed at the follow-ups determined by the study protocol.”

## Discussion

Degenerative meniscus tears are common and related to the development of knee osteoarthritis [1,3-5]. Arthroscopic partial meniscectomy is the current treatment of choice in patients with mild or no concomitant knee osteoarthritis but this has not been formally evaluated in randomized placebo controlled trials.

The outcome of this study will show whether arthroscopic partial meniscectomy is a viable treatment modality. Inclusion of a sham surgery treatment will enable us to study the effect of the partial meniscectomy per se. The findings of this research will potentially be of international importance and will be readily translatable into clinical practice, irrespective of the results. If our results are in favor of APM, we will have evidence to support continued use of APM in this patient category. If, on the other hand, our results indicate that the efficacy of APM is less than placebo (and it may do more harm), then this would also significantly impact upon current practice and APM should not be the treatment of choice in middle-aged patients with an MRI-verified meniscus tear and mild or no knee osteoarthritis. No difference between APM and placebo might not be regarded as a strong enough piece of evidence to stop operating on middle-aged patients with meniscus tear and mild OA. However, a finding of superiority for arthroscopy would certainly increase the tendency to treat these patients surgically, and a finding of superiority for placebo would discourage operative treatment. Finding no effect of APM would support the notion that a degenerative meniscus tear is the first sign of future knee OA. If so, the treatment of choice should conform to treatment guidelines for mild and moderate knee OA.

The study design has some limitations. The surgeon’s level of experience may differ since we need to allow general orthopedic surgeons to operate and not only sports surgeons. However, this has the benefit of an increased external validity. The population in the study is somewhat heterogeneous, from patients with no osteoarthritis to patients with mild osteoarthritis. We do not know whether a meniscus tear has different etiology in those with and without radiographic OA and how this may affect the result. There is no consensus on what defines a symptomatic meniscus tear or whether or not to perform an MRI before surgery. Clinical tests (McMurray, Apley, etc.) have not been proven to diagnose a meniscus tear accurately [34]. Therefore in this study, we included patients with knee pain and an MRI-confirmed medial meniscus lesion but there is a risk that, in some patients, symptoms may actually not be caused by the meniscus tear. Further, other patients who would otherwise have undergone a knee arthroscopy may be excluded due to the MRI not confirming a suspected meniscus tear. Another limitation of using MRI as a diagnostic tool is the risk of a false positive result. If the patient will be randomized to a sham operation, this error will never be discovered. We chose not to perform a diagnostic arthroscopy in the sham group primarily to reduce the risk of deep infection which we find would be unacceptable for a sham intervention. Other reasons were to avoid any accidental osteochondral lesions from the arthroscope and unwanted intervention from the surgeons.

The study does not include an activity score. A literature search revealed a lack of valid self-reported instruments of activity level for this diverse middle-aged population of varying physical activity levels. Since providing patients with accelerometers was not an option due to logistic reasons, we have not included any measure of activity level in this trial.

In spite of the above limitations, this study has strong methodological rigor through its design as a double-blinded placebo controlled RCT, compared to the earlier non-controlled studies of degenerative medial meniscus tears.

Ethical considerations are important when performing a surgical placebo controlled study. One may ask, ‘Is it ethical to perform placebo-controlled RCTs of surgery?’ since the initial precept in medicine is “First, do no harm”. An equally valid question though, may be, ‘Is it ethical not to perform placebo-controlled RCTs within orthopedics [35] and instead, potentially perform under-researched operations which may not benefit the patient, or worse, do harm?’

A recent study of vertebroplasty [36,37] has effectively shown how a placebo-controlled surgical trial can evaluate a given procedure that has been adopted widely despite an absence of robust evidence. Currently, there are three reasons to perform yet another placebo-controlled arthroscopy study, in addition to the one performed by Moseley and collaborators (2003). Firstly, the current study will focus on younger patients; secondly, these patients are at an earlier stage of disease and have not yet developed severe knee OA; and thirdly, a replication study is required to make Moseley’s evidence more convincing.

## Abbreviations

OA: Osteoarthritis; APM: Arthroscopic partial meniscectomy; RCT: Randomized controlled trial; KOOS: Knee injury and osteoarthritis outcome score; PRO: Patient-reported outcome; QOL: Quality of life

## Competing interests

The authors declare that they have no competing interests.

## Authors’ contribution

KH, SL and ER participated in the conception and design of the study. KH will participate in the recruitment of participants. KH, SL, RC and ER were involved in drafting the manuscript or revising it, all authors read, commented, and approved the manuscript.

## Authors’ information

KH: MD, PhD student, Research Unit for Musculoskeletal Function and Physiotherapy at the Institute of Sports Science and Clinical Biomechanics, University of Southern Denmark, Odense, Denmark; SL: MD, PhD, Professor at the Department of Orthopedics, Lund University, Sweden, Research Unit for Musculoskeletal Function and Physiotherapy at the Institute of Sports Science and Clinical Biomechanics and Department of Orthopedics and Traumatology, University of Southern Denmark, Odense, Denmark; RC, MSc, PhD, Associate Professor of Statistics in Medicine, Musculoskeletal Statistics Unit, the Parker Institute, Department of Rheumatology, Copenhagen University Hospital, Frederiksberg, Copenhagen F, Denmark; ER: PT, PhD, Professor and Head of Research Unit for Musculoskeletal Function and Physiotherapy at the Institute of Sports Science and Clinical Biomechanics, University of Southern Denmark, Odense, Denmark.

## Pre-publication history

The pre-publication history for this paper can be accessed here:

http://www.biomedcentral.com/1471-2474/14/71/prepub
